# AC and Phase Sensing of Nanowires for Biosensing

**DOI:** 10.3390/bios6020015

**Published:** 2016-04-19

**Authors:** Marco Crescentini, Michele Rossi, Peter Ashburn, Marta Lombardini, Enrico Sangiorgi, Hywel Morgan, Marco Tartagni

**Affiliations:** 1 Department of Electrical, Electronic and Information Engineering, University of Bologna, Cesena Campus, Cesena 47521, Italy; marco.crescentini3@unibo.it (M.C.); michele.rossi@unibo.it (M.R.); enrico.sangiorgi@unibo.it (E.S.); 2 School of Electronics and Computer Sciences, and the Institute for Life Sciences, University of Southampton, Southampton SO17 1BJ, UK; pa@ecs.soton.ac.uk (P.A.); ml09v@ecs.soton.ac.uk (M.L.); hm@ecs.soton.uk (H.M.)

**Keywords:** nanowires, nanosensors, phase detection, impedance spectroscopy, AC sensing

## Abstract

Silicon nanowires are label-free sensors that allow real-time measurements. They are economical and pave the road for point-of-care applications but require complex readout and skilled personnel. We propose a new model and technique for sensing nanowire sensors using alternating currents (AC) to capture both magnitude and phase information from the sensor. This approach combines the advantages of complex impedance spectroscopy with the noise reduction performances of lock-in techniques. Experimental results show how modifications of the sensors with different surface chemistries lead to the same direct-current (DC) response but can be discerned using the AC approach.

## 1. Introduction

The development of sensitive and specific biosensors remains a significant challenge. Among many issues, one major problem is the miniaturization and efficiency of the readout electronics. Silicon nanowires (Si-NWs) and nanoribbons have been proposed as for a new class of biosensors, primarily because they are label-free sensors and allow real time measurements [1,2,3], even in complex physiological media [4,5,6].

Nanowire sensors are very low cost (disposable) sensors since fabrication can be performed using standard top-down processes, based on mature photolithography, thin film deposition and plasma etching [7,8,9,10]. However, a typical readout system is bulky and expensive requiring a complex setup and skilled personnel. To enable the routine use of biochemical sensors, they will have to be simple and low cost, so that they can be used in point-of-care locations other than clinical laboratories. To address this need, integrated sensor systems are emerging that can be used within portable systems [11,12], [13,14,15] where the sensor chips are fused with custom electronics into a user-friendly package.

Nanowire and nanoribbon sensing is usually performed with simple DC measurements [16,17,18,19] although alternating currents (AC) characterization of the electrical properties of nanowires (NWs) is a standard procedure [20,21,22]. In the last few years, nanowire AC sensing has become an important technique which has advantages with respect to DC sensing [23]. However, the majority of the literature describes AC sensing based on single-phase lock-in detection, where only the magnitude of the impedance, or power response of the NW (or carbon nanotube), is investigated [24,25,26]. Only a few recent manuscripts describe NW complex AC sensing, measuring the analyte with both magnitude and phase of the impedance [27,28].

In this paper, we investigate the potential of complex AC sensing of Si-NWs to provide information on magnitude and phase of the impedance. Since the exact response of the nanowire sensors is not fully understood, it is possible that measurement of the complex NW impedance could provide more information on the binding events occurring at the interface, including time-dependent behavior of local charges. DC sensing of NWs is based on modulation of the channel resistivity due to changes in the surface potential. Nanowires can also be viewed as a distributed line with the resistance and capacitance per unit length determined by the concentration of target molecules. In particular, NW capacitance could reveal further information concerning the target molecules, which could only be sensed by AC methods since AC methods are sensitive to the displacement of charge at the interface. Therefore, the development of a compact and precise readout system capable of measuring the complex AC impedance of nanowires is an important step for testing the dynamic properties of these nanosensors.

This paper describes an AC sensing system based on a dual-phase lock-in technique. Impedance spectrometry is used to measure NW characteristics. Results reveal how changing the surface charge on the nanowire leads to the same DC response but different AC impedance properties. Section 2 provides a full description of the entire setup, from fabrication and physical behavior of nanowires to implementation of electronic circuits and microfluidic of the AC sensing system. This section also provides the motivation to the AC approach. Finally, Section 3 describes a simple electrical model for the NW and compares it with experimental DC and AC measurements.

## 2. Materials and Methods

### 2.1. Device Under Test

The silicon nanowire chips (14.8 × 14.8 mm^2^) contain 8 sets of p-type top-down fabricated nanowires [7]. The devices were fabricated using a low-cost photolithography process, based on thin film technology and a dry spacer etching technique. Such devices are suitable for low cost mass production.

**Figure 1:**
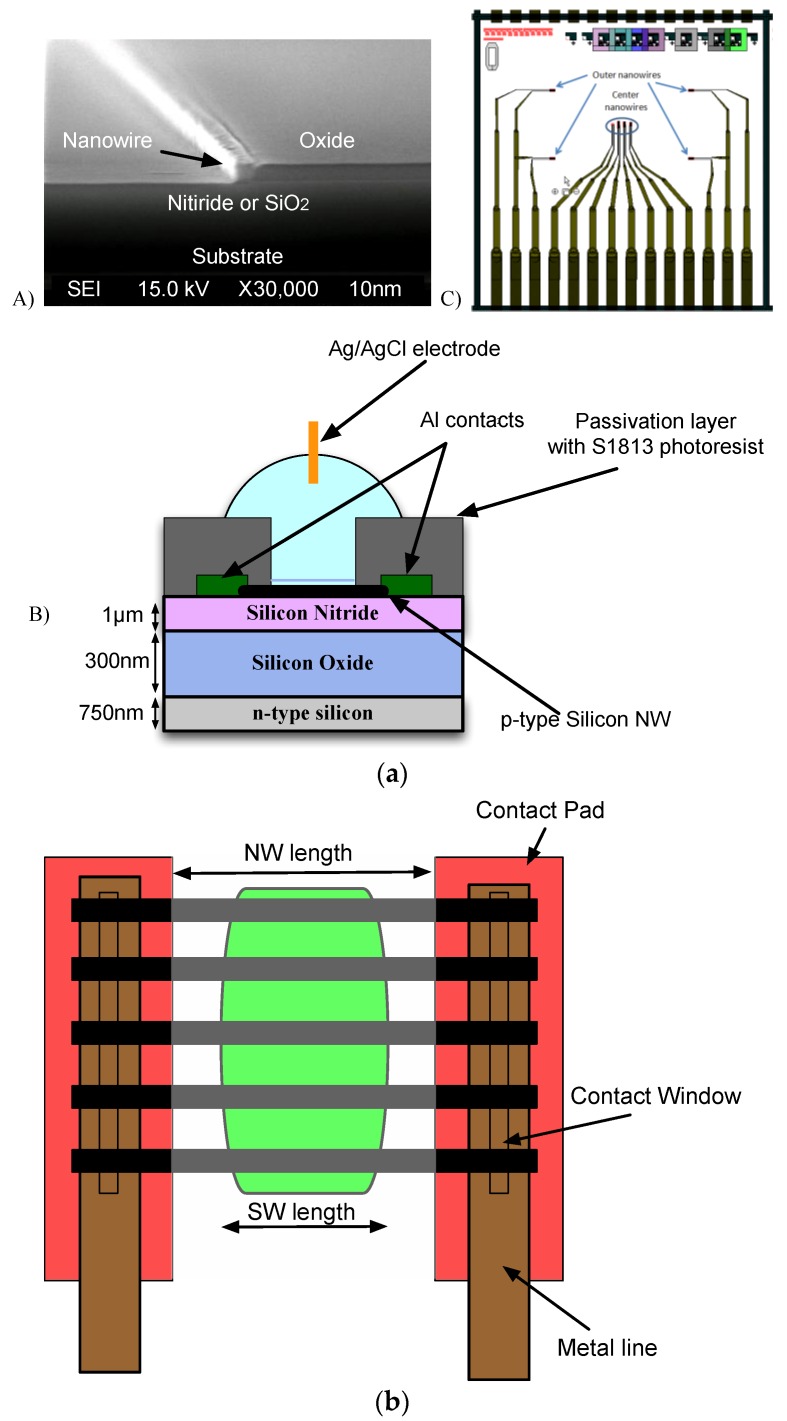
(**a**) Cross section of the nanowire: (**A**) SEM image of a fabricated polysilicon nanowire; (**B**) cross sectional diagram showing the different layers; (**C**) Layout of a NW chip showing connection pads compatible with standard commercial connectors; (**b**) Top view of a single NW device. Different numbers of nanowires (from 10 to 320) are connected in parallel depending on the nanowire set. The green area is the sensing window. The remainder of chip is covered with a layer of photoresist. The red area is the source/drain pads.

The nanowires are 40 µm long with a rectangular cross section of 100 nm × 100 nm. They are arranged in sets. The outer four sets have the same number of parallel nanowires (30); while the inner sets have a variable number of parallel nanowires (10 to 320), see Figure 1a.

Figure 1a shows the device cross section. It consists of an n-type silicon substrate, 750 nm plasma enhanced chemical vapor deposited (PECVD) oxide layer, and a 300 nm silicon nitride layer. Oxide pillars are created by PECVD and patterned using photolithography and anisotropic etching. On those pillars a 100 nm amorphous silicon (α-Si) film is deposited by low-pressure chemical vapor deposition (LPCVD) at 560 °C and then doped by boron implantation at a dose of 1 × 10^18^/cm^2^ and an energy of 25 keV. Rectangular-shaped nanowires are formed using a special anisotropic dry-etching process performed using an Oxford Instruments Plasma Technology 80+ reactive ion etcher (RIE) system at 160 W input power, with a SF_6_ flow of 12 sccm, an O_2_ flow of 12 sccm and a pressure of 30 mTorr.

**Figure 2:**
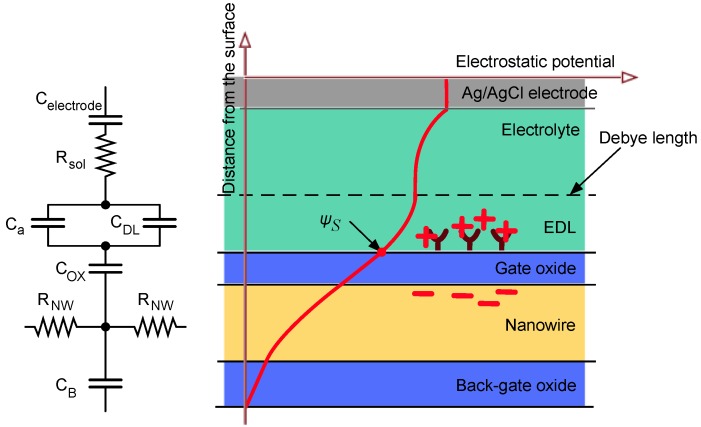
The solid-liquid interface showing the electrical double layer (EDL), the Debye length and electrostatic potential over the entire structure (from electrolyte to nanowire). A simplified version of the equivalent electrical model given by [34,35] is shown on the left, for the case of a small surface potential. *C_DL_*, *C_A_*, *C_OX_*, *C_B_* are the double layer, surface chemistry, oxide, and bulk capacitances, respectively. *R_SOL_* is the solution resistance and *R_NW_* is the NW resistance.

At the end of the process, a gate oxide, nominally 10 nm thick, is grown at 900 °C creating a stable surface for nanowire sensing in liquid. Finally, aluminum contacts were made at both end of the polysilicon nanowire by the creation of heavily doped (with a dose of 1 × 1021/cm^2^) source/drain pad regions, highlighted in red in Figure 1b. The sensing window (green in Figure 2b) over the nanowire is the only exposed region, while the rest of the NW is covered with layer of photoresist.

Metal pads close to the lower scribe lines (Figure 1a) provide contacts and have a pitch of 1 mm, compatible with standard commercial connectors (Samtec SEI series). This simplified connection to a PCB allowing different NW chips to be tested.

### 2.2. NW Physical Model

A silicon NW senses molecules based on modulation of its conductance by a change in the charge density at the interface, similarly to an ion-sensitive field effect transistor (ISFET). The gate-sensing layer separates the nanowire oxide from the aqueous electrolyte [29,30,31].

Any free charge in the system, electrons/holes in semiconductors and ions in solution, are subject to the same laws: Electrostatic forces and the statistical distribution of charges. Therefore the charge distribution can be determined from the electrostatic potential profile given by Poisson’s equation, and the distribution of charges given by Boltzmann statistics.

Assuming a vertical cross section divided into electrically independent parts (electrolyte, gate oxide and semiconductor), the free carriers in each section have a non-uniform distribution given by a solution to the Poisson-Boltzmann equation. The solution to this non-linear equation gives the exact profile of the potential: for example in the electrolyte the potential decays exponentially from the surface. This behavior is called electrical double layer (EDL) and the decay distance is the Debye length λ*_D_* [32]. The differential capacitance per unit area of the double layer is:

(1) $$C_{DL}=\frac{d\sigma }{d\psi_{S}}≅\frac{\varepsilon_{0}\varepsilon_{r}}{\lambda_{D}}$$

where σ is the surface charge density, and ψ*_S_* is the electrostatic potential at the interface. The Debye length is given by:

(2) $$\lambda_{D}=\sqrt{\frac{\varepsilon_{0}\varepsilon_{r}kT}{2z^{2}q^{2}n_{0}}}$$

where *z* is the ionic valance and *n*_0_ is the ion concentration in the bulk. The charge density of the semiconductor, and thus the conductance of the NW, is non-linearly related to the electrostatic potential ψ*_S_* at the interface as shown in [33] (Figure 2). The Poisson-Boltzmann model does not take into account the contribution of the charge equilibrium at the oxide-electrolyte interface. This effect, when added to the model, is called the site-binding model [33], where typically binding occurs at a range shorter than the Debye length. More specifically, the surface of any oxide consists of hydroxyl groups (silanol groups Si-OH for silicon dioxide) that may be protonated or deprotonated depending on the pH of the solution and on the isoelectric point (IEP) of the surface, resulting in a positively or negatively charged surface according to:

(3) $$\begin{array}{l} SiOH_{2}^{+}↔SiOH+H^{+}\\ SiOH↔SiO^{-}+H^{+} \end{array}$$

If the pH of the solution is higher than the IEP then the surface is deprotonated and acts as if negative charges have been added to the surface, increasing the concentration of the holes and therefore the nanowire conductance. The opposite occurs if the pH is lower than the IEP.

From the viewpoint of this model, the equilibrium constants need to be included to complete the description with respect to H^+^ concentration. The model is equivalent to that derived for ISFETs by Bergveld in 1970 [34]. A detailed description of the site-binding model is found in [35]. Cui *et al.* showed how nanowires can be highly sensitive pH sensors [36,37].

On the base of the above we expect the following:

- The concentration of the solution modulates the Debye length according to Equation (2), thus changing the exponential decay of the electrostatic potential (Figure 2, right), modeled as the double layer capacitance *C_DL_*.
- Any charge variation due to site-binding (such as pH and molecular interactions at the interface) occurring within the Debye length directly influence the potential profile at the interface according to Poisson’s equation. The concentration of hydrogen ions is usually much smaller than the concentration of salt ions; therefore these play a negligible role in determining the Debye length.
- The functionalization of the NW surface induces, together with local electrostatic potential variation, charge dynamic effects that can be modeled as a differential capacitance *C_A_* in parallel with the double layer capacitance [33].

An electrical model of the NW interface together with a cross-section is illustrated in Figure 2. The distributed model that will be shown in Section 3 is based on the above model. In terms of sensing, DC measurements can measure variations in *R_NW_* (mainly related to site-binding effects), whilst AC sensing can measure, in addition to *R_NW_*, *C_DL_* and *C_A_*, giving a deeper understanding of the physical phenomenon acting on the surface, hence a different sensing mechanism.

### 2.3. AC Nanowire Sensing

The impedance of nanowires depends on time-dependent variations in the electrical potential, as given by equation (1) *and/or* by changes in bound charges.

**Figure 3:**
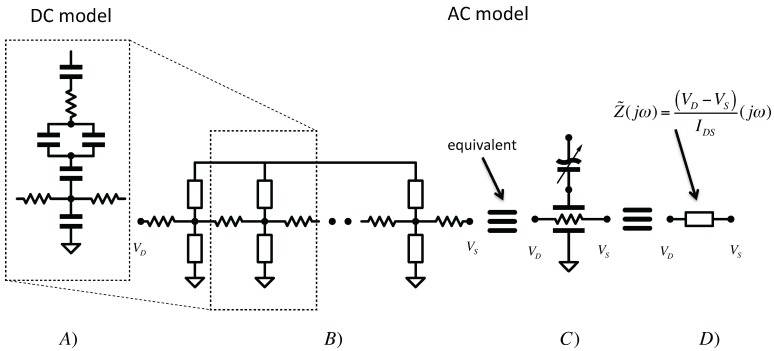
(**A**) DC-sensing model described in previous section; (**B**) AC-sensing model defined by composing a distributed model element based on previous one; (**C**) Compact representation of the AC-sensing model; (**D**) Complex impedance sensed in or approach.

On the basis of the DC-sensing simplified model reported in Figure 2, the AC sensing could be defined by an infinite number of DC-sensing stages (Figure 3A) placed side-by-side as illustrated in Figure 3B describing a distributed element model shown in Figure 3C. Therefore, our approach is sensing the equivalent impedance illustrated in Figure 3D. The accuracy of this model using SPICE simulations with respect to experimental results will be shown in the next section.

**Figure 4:**
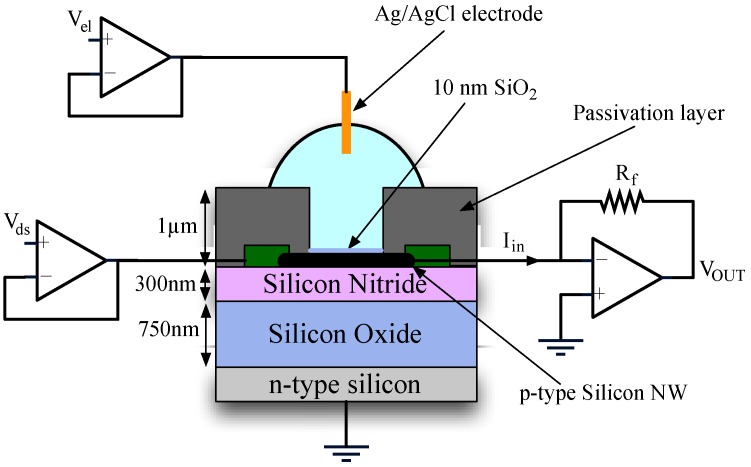
Typical experimental setup with the buffer potential fixed with a liquid gating electrode and substrate connected to ground. A stimulus voltage is applied at the source and a current read by a transimpedance amplifier at the drain.

There are different ways of applying AC signals across a nanowire and a typical single-ended architecture is shown in Figure 4. The exact way in which the AC signal is applied depends on the static characteristic of the device. The use of small *V_gs_* or *V_ds_* signal is interchangeable and depends on the chosen bias conditions. First, we characterized the nanowire in the *I_d_(V_ds_, V_gs_)* plane, noting that for small *V_gs_* (*i.e.*, *V_gs_* ≤ 100 mV) the device works in a strong inversion, linear region. Therefore, for this bias condition, the sensitivity of the drain current to *V_ds_* changes is stronger than for changes in *V_gs_*. Therefore, it is preferable to apply the signal to the source/drain terminal with the bulk set to ground. This approach is preferable for impedimetric sensing since the voltage and the current are applied to the same terminal, thus avoiding phase error. A small sinusoidal voltage (100 mV) is applied to one end of the nanowire while a current is measured at the other. The same scheme could be used for DC mode simply using an offset bias on one side of the NW.

### 2.4. Impedance Lock-In Principle

**Figure 5:**
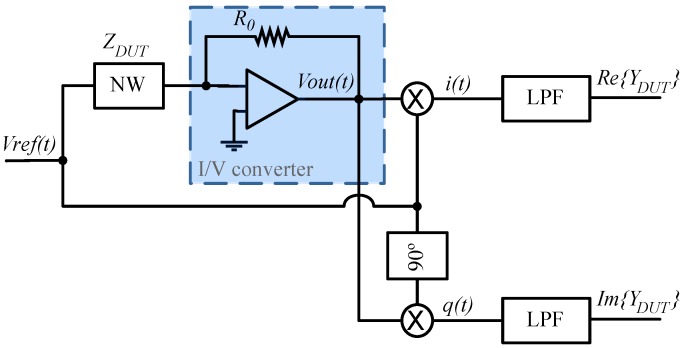
Scheme describing a lock-in technique.

The dual-phase lock-in is a phase sensitive detector that measures amplitude and phase of an AC signal in a noisy environment. The operating principle is depicted in Figure 5. A sinusoidal stimulus:

(4) $$V_{ref}\left(t\right)=V_{0}\cos\left(\omega_{0}t\right)$$

is applied to a device under test (DUT) and the current is converted into an output voltage:

(5) $$V_{out}\left(t\right)=\frac{V_{ref}}{Z_{DUT}}R_{0}=\frac{V_{0}}{\left|Z_{DUT}\right|}R_{0}\cos\left(\omega_{0}t-\theta_{DUT}\right)$$

where *R*_0_ is the transresistance of the I/V converter, *Z_DUT_* is the complex DUT impedance and θ*_DUT_* is the phase contribution of *Z_DUT_*.

Both voltages, *V_OUT_* and *V_REF_*, are fed into a phase-sensitive detector where *V_OUT_* is multiplied by the reference voltage and the 90° shifted version, providing an in-phase signal *i* and quadrature signal *q* as follows:

(6) $$\begin{array}{ll} i & =V_{OUT}\left(t\right)\cdot V_{0}\cos\left(\omega_{0}t\right)=\frac{V_{0}^{2}}{\left|Z_{DUT}\right|}R_{0}\cos\left(\omega_{0}t-\theta_{DUT}\right)\cos\left(\omega_{0}t\right)\\ & =\frac{V_{0}^{2}}{2\left|Z_{DUT}\right|}R_{0}\left[\cos\left(-\theta_{DUT}\right)+\cos\left(2\omega_{0}t-\theta_{DUT}\right)\right] \end{array}$$

(7) $$\begin{array}{ll} q & =V_{OUT}\left(t\right)\cdot V_{0}\sin\left(\omega_{0}t\right)=\frac{V_{0}^{2}}{\left|Z_{DUT}\right|}R_{0}\cos\left(\omega_{0}t-\theta_{DUT}\right)\sin\left(\omega_{0}t\right)\\ & =\frac{V_{0}^{2}}{2\left|Z_{DUT}\right|}R_{0}\left[\sin\left(2\omega_{0}t-\theta_{DUT}\right)-\sin\left(-\theta_{DUT}\right)\right] \end{array}$$

Low-pass filters remove the high frequency terms in Equations (6) and (7), giving the real and imaginary parts of the DUT admittance:

(8) $$ℜe\left\{Y_{DUT}\right\}\sim \frac{R_{0}V_{0}^{2}}{2}\left|Y_{DUT}\right|\cos\left(-\theta_{DUT}\right)$$

(9) $$ℑm\left\{Y_{DUT}\right\}\sim -\frac{R_{0}V_{0}^{2}}{2}\left|Y_{DUT}\right|\sin\left(-\theta_{DUT}\right)$$

### 2.5. Circuit Implementation

Typical nanowire resistance is in the range of 100 kΩ to 100 MΩ [5,6,8,19,38,39,40] so that the currents are of the order of tens to hundreds of nA. Our measurement system is a portable test board that implements a two electrode potentiostat with a lock-in measurement technique for complex impedance detection as described in previous section [41].

The board is based on the AD630 switched demodulator [42]. A transimpedance input amplifier converts the current signal from the sensors prior to the phase-sensitive detection. The signal is pre-amplified by an AD822 which shows low-power consumption, rail-to-rail, low noise operating performance (13 nV/√Hz @ 10 kHz), and low input bias current [43].

**Figure 6:**
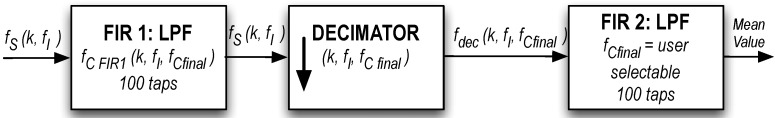
Representation of the adaptive two stage low pass FIR filter implemented in the LabView interface.

The signal conditioning process is shown in Figure 6, and has 3 stages: First two stages consist of a finite impulse response digital low-pass filters using a FIR Kaiser window and a decimator. Weight taps were calculated with MATLAB to provide the best configuration for all the required frequencies.

Filtering and data manipulation are performed in the digital domain, providing a particularly flexible system, with a simple analog front-end that can be used for different applications. An auxiliary potential control circuit applies a back-gate and/or liquid gate potential.

### 2.6. Microfluidics

**Figure 7:**
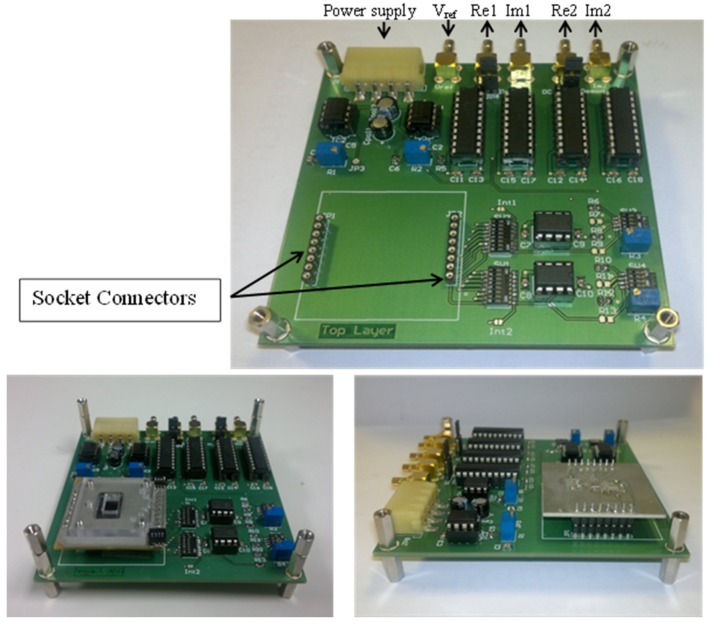
Pictures of the signal acquisition system.

**Figure 8:**
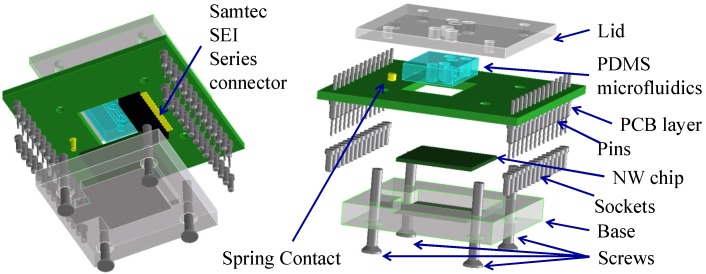
Drawings of the nanowire chip holder highlighting the different components stacked in a “sandwich approach”.

The board is designed to be modular and has a single electronic readout interface. Different sensors can be connected using a socket (Figure 7). A custom microfluidic board (4 × 4 cm^2^) interfaces to the nanowire chip [7]. The microfluidic interface consists of four parts (Figure 8):

- A base, which holds the NW chip and makes contact to the back gate with a small piece of aluminium foil providing. A recess enables automatic alignment of the contact pads of the NW chip with the connector.
- A windowed two-layer PCB, with Samtec**^®^** SEI connector, a spring connector and two 4-way surface mount device (SMD) switches. The spring connector contacts the aluminium foil base. The 4-way SMD switches connect the *V_REF_* signal to the selected Si-NWs under test.
- A rectangular PDMS microfluidics gasket, designed and moulded as required (e.g., with or without microchannels for solution flow), ensures a tight seal between the sensitive NW chip surface and the bottom layer of the PCB avoiding electrolyte leakage;
- A lid to package the microfluidic gasket as well as the whole device.

## 3. Results and Discussion

### 3.1. Equivalent Model

**Figure 9:**
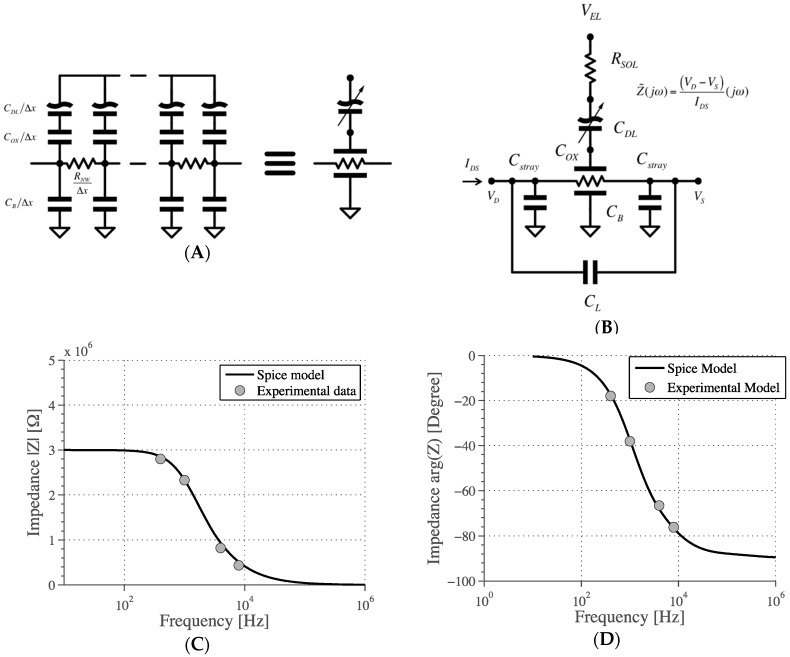
Distributed model of the nanowire channel (**A**) and AC sensing model (**B**). Comparison of the SPICE model with experimental data (bare nanowires) in impedance module (**C**) and phase (**D**). *R_NW_* = 3 MΩ, *C_L_* = 5 pF, *C_stray_* = 30 pF, *C_S_* = *C_OX_//C_DL_* = 15 pF, *C_B_* = 100 fF, *R_SOL_* = 2 MΩ.

In order to understand the complex impedance behavior of the device, an electrical model of the nanowire is proposed in Figure 9. This is a distributed network composed of a series of identical RCR T-network elements surrounded by lumped parasitic capacitances. The AC excitation consists in applying a sinusoidal voltage (100 mV) across the NW and thus measuring the corresponding device current. However, the device is characterized by a distributed RC time constant, as shown in Figure 9A, given by the channel resistance *R_NW_* and channel capacitances. On the solution side we have the double layer capacitance *C_DL_* and the thin oxide capacitance *C_OX_*, whilst on the other side we have the bulk capacitance *C_B_*. Since the excitation frequency is comparable to the reciprocal of the time constant, we cannot model the device with a lumped RC circuit. Therefore, in our approach we model the device with a distributed circuit (50 sections) as sown in Figure 9A. The model has been simulated with SPICE in order to determine the relationship between the amplitude and the current that is described by the complex impedance $\tilde{Z}(j\omega )$ and compare it with the experimental results. The whole device is shown in Figure 9B.

To setup the model, the parasitic capacitance of the contacts was measured for several different nanowire chips using a probe station (Cascade Microtech prober, associated with Agilent 4279A 1 MHz CV meter) both in air and buffer conditions. Values range from 2 to 5 pF for the track-track capacitance (*C_L_*) and from 18 to 30 pF for the track-bulk capacitance (*C_stray_*).

Assuming the NW works in the strong inversion regime, it can be modeled as a variable resistor modulated by surface charges neglecting the semiconductor depletion capacitance, usually taken into account in modelling FET devices [2], so that the only variable is the capacitive contribution from the semiconductor interface (*C_S_*) [14]. This surface capacitance *C_S_* is the series combination of the oxide capacitance *C_OX_*, (the SiO_2_ layer on top of the NW), and the electrical double layer capacitance *C_DL_* as illustrated in Figure 9A. When the surface oxide is changed by chemical modification, an additional capacitance *C_A_* is added in parallel with *C_DL_* in the model, as explained in [32]. Value of *C_OX_* has been calculated from geometry and physical parameters, while *C_DL_* was estimated from experimental data.

The charge transfer resistance usually considered in electrode-electrolyte interface model [15,16] can be neglected in the absence of Faradaic reactions. This is confirmed by measurements of the current between the liquid gate electrode and the nanowire drain contact, which is <10 pA. The impedance of the electrode/electrolyte interface [31,32] at the liquid gate electrode was also neglected due to its very large dimension compared to the nanowires. It is modeled as an ideal electrode and sets the potential of the buffer solution.

Complex impedance measurements of bare nanowires immersed in pH 7 phosphate buffer were fitted to this circuit. As shown in Figure 9C,D, the equivalent model matches the data with the following parameters: *R_NW_* = 3 MΩ, *C_S_* = *C_OX_//C_DL_* = 7.5 pF, *C_DL_* = 74 *pF*, *C_L_* = 5 pF, *C_B_* = 100 pF, *C_stray_* = 30 pF, *R_SOL_* = 2 MΩ. Note that according to the graphs, the AC magnitude sensing follows the DC behavior at low frequency whilst at higher frequency it is attenuated by a factor depending on the frequency, as in a dominant pole behavior. Note also in Figure 9D that, as long as the frequency is very low, the voltage and the current are in-phase as in a pure resistor, whilst for high frequency the voltage and the current are in quadrature phase-lag, as expected.

### 3.2. AC Measurements

**Figure 10:**
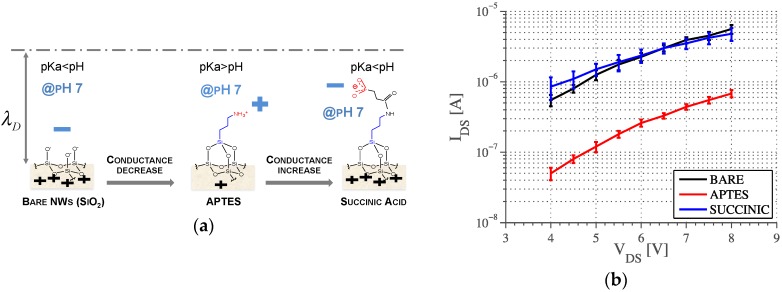
(**a**) Change in the surface charge at pH = 7 for different surface chemistry. The surface is deprotonated for both bare and succinic treated NWs while it is slightly protonated for *APTES* treated NWs; (**b**) DC measurements on NWs with different surface chemistry, pH = 7.

The surface of the nanowires were modified using two different chemistries: (i) vapor deposition of 3-aminopropyltri-ethoxysilane (*APTES*); and (ii) further exposure to *N*-(3-Dimethylaminopropyl)-*N*′-ethylcarbodiimide hydrochloride, *N*-hydroxysulfosuccinimide sodium salt and succinic acid. These two methods produce surfaces with different surface charge and dissociation constants (pKa) as confirmed by DC measurements at fixed pH 7. The nanowire conductivity decreases after *APTES* functionalization due to the presence of the amino group (pKa~9) that protonates at pH 7, reducing the local concentration of holes (carriers) in the p-type nanowire [26]. Conversely, it increases with the succinic acid, since the terminal carboxyl group (pKa~5) deprotonates at pH 7, resulting in a conductance similar to the bare nanowires, as previously reported [7] (Figure 10). The DC results presented in Figure 10b show how the behavior of the *bare* and *succinic* functionalized wires are similar.

**Figure 11:**
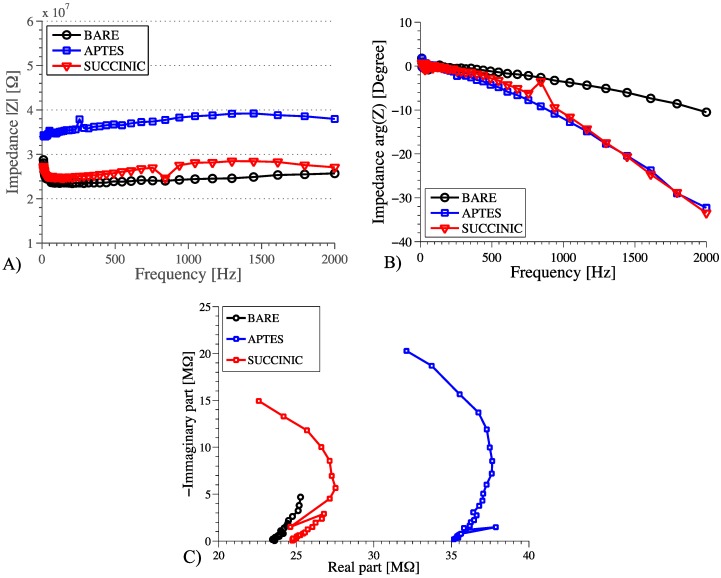
(**A**) AC impedance magnitude and (**B**) phase measurements showing how different surface chemistries can be discriminated. Simple DC measurements show no difference; (**C**) Cole-Cole plot of the same data.

These DC results were confirmed using AC sweeps of nanowires immersed in 1 mM phosphate buffer solution at pH 7 (Figure 11). For these experiments the custom acquisition board was used, as described in section II. This board can work up to 2 kHz; which is low enough to observe all the dynamics involved in NW sensing, and high enough to demonstrate AC phase sensing. The impedance magnitude is in agreement with the DC measurements confirming the expected behavior of the modulation of conductivity in response to changes in surface charge. The phase plot shows that the *APTES* and *succinic* *acid* nanowires have similar properties, but different to bare nanowires. This could be explained by a variation in the site-binding capacitance *C_A_*. We summarize the behavior as follows:

- The effect of the charge change following functionalization changes the NW resistance, thus the magnitude of the AC impedance is similar to the DC (*bare* NW and *succinic acid* showed the same behavior);
- Surface chemistries with similar effects on NW charge have different properties that can be measured by phase detection and modeled through different values of *C_A_*, which should be added in parallel with C_DL_ in the model, as explained in [32].

The behavior can also be represented with a Cole-Cole plot shown in Figure 11C. The *bare* and *succinic* response have the same magnitude and the same intercept at f = 0 Hz (as in DC) whilst *APTES* has larger magnitude values. On the contrary, *succinic* and *APTES* have the same phase behavior whilst *bare* NWs show a much smaller phase shift.

## 4. Conclusions

Nanowire and nanoribbon sensing is usually performed with either quasi-static measurements (DC) or single-phase lock-in. Those only probe changes in the surface charge on the NW conductance. In this paper we introduce AC phase sensing of nanowires. A compact AC impedance system was developed based on a lock-in technique. pH measurements of different nanowires were used to compare AC sensing against standard DC sensing. The system demonstrates the ability of AC sensing to discriminate between different surface chemistries that are indistinguishable by DC measurement. An equivalent electrical circuit of the nanowire has been proposed to model the complex impedance of the nanowire device.

## Abbreviations

The following abbreviations are used in this manuscript:

AC	Alternating Current
DC	Direct Current
NW	Nanowire
CMOS	Complementary Metal Oxide Semiconductor
Si-NW	Silicon Nanowire
PECVD	Plasma Enhanced Chemical Vapor Deposited
LPCVD	Low-Pressure Chemical Vapor Deposition
RIE	Reactive Ion Etcher
PCB	Printed Circuit Board
ISFET	Ion-Sensitive Field Effect Transistor
EDL	Electrical Double Layer
IEP	Isoelectric Point
DUT	Device Under Test
SMD	Surface Mount Device
PDMS	Polydimethylsiloxane
APTES	3-aminopropyltri-ethoxysilane
